# Effect of Pore Size of Porous-Structured Titanium Implants on Tendon Ingrowth

**DOI:** 10.1155/2022/2801229

**Published:** 2022-04-25

**Authors:** Yupeng Guo, Fei Liu, Xuting Bian, Kang Lu, Pan Huang, Xiao Ye, Chuyue Tang, Xinxin Li, Huan Wang, Kanglai Tang

**Affiliations:** Department of Orthopedics/Sports Medicine Center, State Key Laboratory of Trauma, Burn, and Combined Injury, Southwest Hospital, Army Medical University (Third Military Medical University), Chongqing 400038, China

## Abstract

**Purpose:**

The reconstruction of a tendon insertion on metal prostheses is a challenge in orthopedics. Of the available metal prostheses, porous metal prostheses have been shown to have better biocompatibility for tissue integration. Therefore, this study is aimed at identifying an appropriate porous structure for the reconstruction of a tendon insertion on metal prostheses.

**Methods:**

Ti6Al4V specimens with a diamond-like porous structure with triply periodic minimal surface pore sizes of 300, 500, and 700 *μ*m and a porosity of 58% (designated Ti300, Ti500, and Ti700, respectively) were manufactured by selective laser melting and were characterized with micro-CT and scanning electron microscopy for their porosity, pore size, and surface topography. The porous specimens were implanted into the patellar tendon of rabbits. Tendon integration was evaluated after implantation into the tendon at 4, 8, and 12 weeks by histology, and the fixation strength was evaluated with a pull-out test at week 12.

**Results:**

The average pore sizes of the Ti300, Ti500, and Ti700 implants were 261, 480, and 668 *μ*m, respectively. The Ti500 and Ti700 implants demonstrated better tissue growth than the Ti300 implant at weeks 4, 8, and 12. At week 12, the histological score of the Ti500 implant was 13.67 ± 0.58, and it had an area percentage of type I collagen of 63.90% ± 3.41%; both of these results were significantly higher than those for the Ti300 and Ti700 implants. The pull-out load at week 12 was also the highest in the Ti500 group.

**Conclusion:**

Ti6Al4V implants with a diamond-like porous structure with triply periodic minimal surface pore size of 500 *μ*m are suitable for tendon integration.

## 1. Introduction

Arthroplasty is an effective treatment for arthropathy, as it can relieve pain, restore physical activities, and improve the quality of life of patients. In prosthetic surgeries involving malignant bone tumors [1], revision arthroplasty [2, 3], periprosthetic fractures, failed osteosynthesis [4], and infections, firm attachment between the tendon and the metal prosthesis is necessary to enhance motor function [5, 6]. However, this challenge has not been solved because tendon tissue has poor vascularity and healing ability [7, 8], and the biocompatibility of prostheses for tissue integration is generally poor. The management of these defects is a challenge for orthopedic surgeons. The 5-year revision-free survival rate following reconstruction of the rotator cuff insertion on a prosthesis is only approximately 50%, which is much lower than that of 89% following intra-articular resection [9]. Shoulder instability after prosthetic reconstruction caused by soft tissue defects accounts for nearly 60%, which is the main cause of replacement failure [5]. The clinical outcome following hip or knee reconstruction of large segmental bone defects can be limited by inadequate reattachment of the soft tissue [10–13].

Titanium and its alloys have been widely used in orthopedic applications [14, 15] for their good mechanical properties and favorable biocompatibility [16]. Titanium itself is bioinert, and polished or abraded titanium has no tissue-bonding ability. Efforts have been made to promote the integration of tendon/ligament and titanium alloy prostheses; for instance, the use of interposed bone or a decalcified bone matrix between the tendon and the prosthesis [17, 18] and biological factors on the surface of the prosthesis have been reported [19]. However, these approaches do not solve certain problems, such as immune rejection, disease transmission, graft nonunion, and the requirement of additional procedures [20].

Porous structures have been recognized as an effective way to eliminate inertia [21], and it has been shown that porous titanium alloys can be effectively integrated with bone in many preclinical [22, 23] and clinical [24, 25] studies. A few studies have been carried out on tendon attachment, which confirmed the promise of porous structures for soft tissue integration [26–29]. Among these studies, however, few are about the accurate pore structures, and none is about the structure correlation between pore and tendons. The optimal pore structure for tendon or ligament ingrowth into porous titanium implants remains unclear.

At the natural insertion of the tendon, collagen fibers are oriented predominantly perpendicular to the interface in tendon, this orientation changes to one that is more oblique when crossing the fibrocartilage region [30], and the boundary line between the soft and hard tissue forms deep interdigitations [31]. Thus, imitating the natural tendon insertion, an appropriate insertion structure should be oblique and interdigitated. Moreover, pores at the insertion should be interconnected to allow cells, nutrients, and oxygen to move into the structure [32] and should have a low curvature, as in animal tissue [33]. Investigations of the most suitable pore sizes are still controversial. Previous studies have identified a range of 300-600 *μ*m that might promote tissue integration [28, 34].

In this study, we manufactured a series of diamond-like triply periodic minimal surface (TPMS) implants of different pore sizes (300, 500, and 700 *μ*m) that are oblique, interdigitated, and well interconnected. We characterized these implants using microfocus X-ray computed tomography (micro-CT) and scanning electron microscopy. Then, we investigated the biological performance of the implants in a rabbit model to examine the fixation ability of each implant into the patellar tendon of the rabbit and to evaluate the tendon tissue ingrowth into the pores and the collagen composition of each implant.

## 2. Materials and Methods

### 2.1. Experimental Animals

The experimental animals were provided by the Animal Center of the Third Military Medical University. A total of 97 two-month-old male New Zealand white rabbits weighing 2.2-2.5 kg were included in the study for tissue section staining and biomechanical tests after implantation of the porous titanium implants. The Laboratory Animal Welfare and Ethics Committee of Third Military Medical University approved all the experimental procedures (AMUWEC20201884).

### 2.2. Preparation of the Porous Titanium Implants

**Figure 1:**
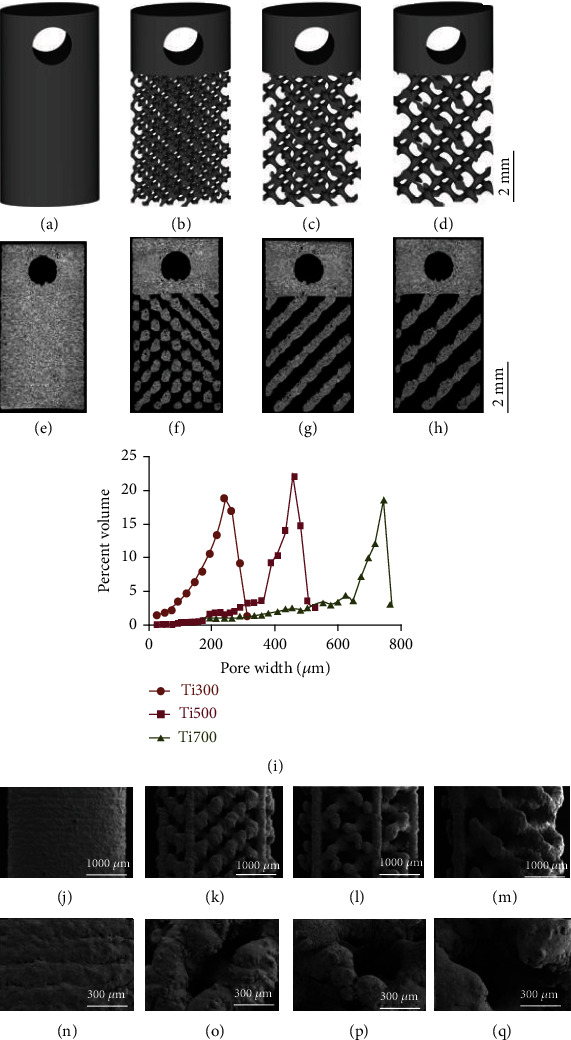
Design and characterization of the titanium implants. (a)–(d) Design of the titanium implants. (a) indicates solid, (b) indicates Ti300, (c) indicates Ti500, and (d) indicates Ti700. (e)–(h) 2D reconstructed image of the titanium implants. (e) indicates solid, (f) indicates Ti300, (g) indicates Ti500, and (h) indicates Ti700. (i) Pore volume distributions in groups Ti300, Ti500, and Ti700. (h)–(q) SEM image of the titanium implants at ×60 (j)–(m) and ×200 (n)–(q). (j) and (n) indicate solid, (k) and (o) indicate Ti300, (l) and (p) indicate Ti500, and (m) and (q) indicate Ti700.

The computer-aided design (CAD) was created using the software MATLAB 2020R (MathWorks, USA) and Magics (Materialise, Belgium). The porous structure cell was designed by the hidden function method and is described by Formula 1 [35]; it is a diamond-like porous structure of TPMSs [36, 37]. By zooming in or out of the structure cells, we could build models with different pore sizes but the same porosity, because the pillars and pores change synchronously when zooming. The STL file of the structure cell was designed by using Magics software, forming the model as shown in Figure 1. The implant is a 2 × 4 × 8 mm elliptical cylinder, and the height of the porous structure is 5.5 mm. The porosity was designed to be 85%. The pore sizes of the implants are 300 *μ*m, 500 *μ*m, and 700 *μ*m (Ti300, Ti500, and Ti700), respectively, and the corresponding diamond-like porous cell sizes are 1 mm, 1.4 mm, and 1.7 mm, respectively. A solid model was designed as the control group (solid) (Figures 1(a)–1(d)).

(1) $$\begin{array}{c} \varphi_{D}\left(x,y,z\right)=\sin\left(x\right)∙\sin\left(y\right)∙\sin\left(z\right)+\cos\left(x\right)∙\sin\left(y\right)∙\cos\left(z\right)+\cos\left(x\right)∙\cos\left(y\right)∙\sin\left(z\right)-0.07\left[\cos\left(4x\right)+\cos\left(4y\right)+\cos\left(4z\right)\right]+1=0. \end{array}$$

Formula 1 shows the description of the porous structure cell.

Gas-atomized, commercially available Ti6Al4V powder (XDM 3D Printing Technology Co., Ltd., Suzhou, China) containing approximately 90.06% titanium with a particle size of 15-53 *μ*m was used as the starting powder. More detailed physical and chemical properties of the Ti6Al4V powder are shown in Table S1.

A selective laser melting (SLM) system (XDM 250; XDM 3D Printing Technology Co., Ltd, Suzhou, China) was used for additive manufacturing (AM) of the porous samples. Porous titanium was manufactured using a laser power of 225 W with a spot size of 70 *μ*m. The laser scanning speed was 1300 mm/s, and the hatching distance was maintained at 120 *μ*m.

After AM was completed, the parts were cooled inside the chamber in an argon atmosphere until the temperature within the powder bed reached 100°C. Then, all samples were removed from the substrate by wire cutting. The heat treatment was intended to relieve the residual stress. Briefly, the samples were slowly heated to 650° in an argon atmosphere and held for 2 h with furnace cooling. Finally, glass beads with particle size of 20-45 *μ*m were used to sandblast porous specimens under the high-pressure gas with 0.5 MPa, the sandblasting time of each porous specimen was about 0.5 minute, and then the specimens were ultrasonically cleaned with pure water for 15 minutes.

### 2.3. Characterization of the Porous Titanium Implants

#### 2.3.1. Porosity

The porosity of the samples was calculated from the weight and apparent volume of the porous elliptical cylinder (2 × 4 × 5.5 mm). All the measurements were conducted 5 times for accuracy.

#### 2.3.2. Microfocus X-Ray Computed Tomography-Based Structural Analysis

Micro-CT-based structural analysis was performed to calculate the porosity and pore size of the samples. The micro-CT system used in the test was a SKYSCAN 1272 (Bruker, Karlsruhe, Germany). The scan was conducted using an accelerating voltage of 90 kV, a beam current of 100 *μ*A, and a filter of 0.11 mm Cu. The rotation step of the sample was set to 0.8°, and 2 frames were taken in each rotation step to reduce random noise. We performed 3D reconstruction with NRecon (Bruker, Rheinstetten, Germany) and obtained reconstructed images with a voxel size of 12 × 12 × 12 *μ*m. These images were processed with CTAn (Bruker, Rheinstetten, Germany). We selected regions of interest (ROIs) close to the edges of each porous sample, and each image inside the ROI was converted to a binary image where the pixel population was assigned to either the foreground (material) or the background (pore). From these data, the pore size, material strut size, and specific surface area of each implant were calculated.

#### 2.3.3. Observation Using Scanning Electron Microscopy

The surface of the porous samples was observed with a Gemini SEM 300 field emission scanning electron microscope (ZEISS, Oberkochen, Germany).

### 2.4. Animal Model and Surgical Procedure

Ninety-seven New Zealand white male rabbits weighing approximately 2.5 kg were included and were randomly divided into five groups. Twenty-three rabbits were divided into each experimental group and underwent implantation of solid, Ti300, Ti500, or Ti700 implants (designated Solid, Ti300, Ti500, and Ti700, respectively). The last 5 rabbits were divided into the control group, which underwent all the procedure steps but with no material implanted.

**Figure 2:**
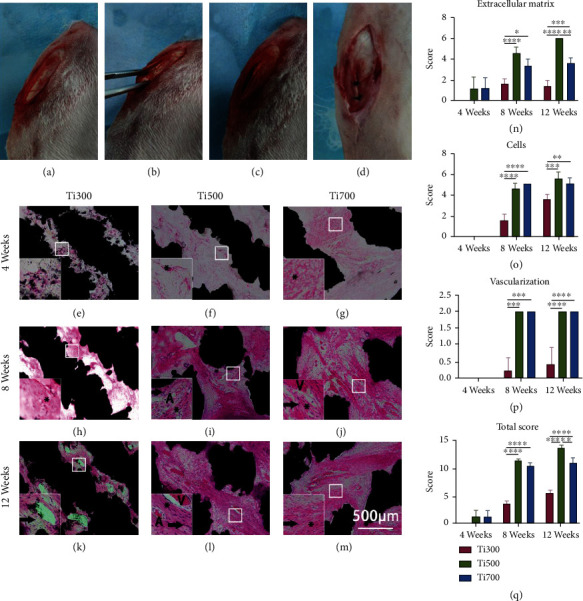
Improvement of histological performance: (a) exposed patellar tendon through paramedian incision, (b) slit in the coronal plane of the patellar tendon, (c) implantation of the titanium material, and (d) suture of the patellar tendon slit. (e)–(m) Representative HE-stained sections of each sample group. (n)–(q) Statistical analysis of histological scores from each group at different time points (*n* = 5; A: artery; V: vein; ^∗^: fiber; arrow: nucleus).

Before implantation, the implants were conventionally sterilized using autoclaving and dried. Intravenous injection of pentobarbital sodium (30 mg/kg) was used for anesthesia. The animals were placed in a lateral position, and the patellar tendon was exposed through a paramedian incision (Figure 2(a)). A slit was made in the coronal plane to form a pocket-like notch (Figure 2(b)), and an implant was inserted into the tendon slit with its hole located proximally (Figure 2(c)). The bilateral edge of the tendon was sutured to prevent implant migration after implantation. Injection of penicillin on postoperative days 1, 2, and 3 was performed on each rabbit to reduce the possibility of infection. The animals were kept individually in cages without immobilization until euthanasia.

At 4, 8, and 12 weeks after implantation, 5 rabbits were euthanized with an overdose of intravenously administered pentobarbital for histological analysis in each experimental group, and 8 rabbits in each experimental group were euthanized for biomechanical testing.

### 2.5. Histomorphometry

Five specimens from each group at each implantation period were prepared for histological examination. After euthanasia by excessive anesthesia, the patellar tendon was harvested with the implant in it. The specimens were fixed in 10% formalin for 7 days and dehydrated in serial concentrations of ethanol (70%, 80%, and 90% for 3 days per concentration and 100% for 3 days two times). The specimens were then embedded in 50% Technovit 7100 polyester resin (Heraeus Kulzer, Hanau, Germany) ethanol solution for 3 days and then embedded in pure Technovit 7100 polyester resin for 3 days two times. Thick sections (150 *μ*m) were cut with an EXAKT 300 hard tissue slicer (EXAKT, Norderstedt, Germany) and ground to a thickness of 40 *μ*m using an EXAKT 400 grinding machine (EXAKT, Norderstedt, Germany). Each specimen was cut into two sections and then stained with hematoxylin-eosin and Sirius red. Histological evaluation was performed on the stained sections using a digital microscope (DSX 500; Olympus Corporation, Tokyo, Japan). Sirius red-stained sections were evaluated under polarized light microscopy.

### 2.6. Biomechanical Tests

**Figure 3:**
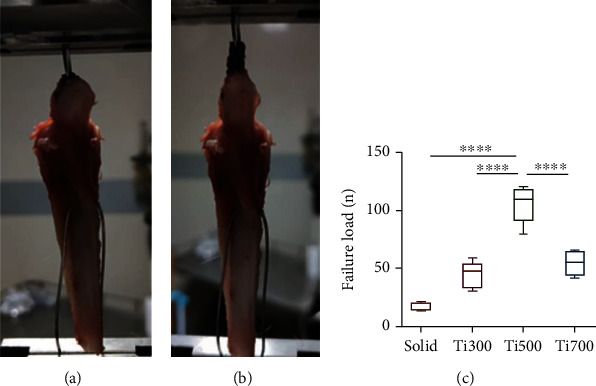
Improvement in biomechanical properties. (a) Fixation of the specimen on a mechanical testing machine. (b) The end point of the test is detachment of the material from the patellar tendon. (c) Statistical analysis of failure loads for each group at week 12 (*n* = 8).

Eight specimens were collected from each group for biomechanical testing at the 12th week after implantation. The specimens were collected right before the biomechanical test and kept on ice being wrapped with normal saline gauze while waiting for the test [38, 39]. Before the test, the patellar tendon was clipped until the hole on the head of the porous implant was completely exposed, and another hole was drilled behind the tibial tubercle. Steel wires were placed through the two holes to fix the specimen on the mechanical testing machine (MTS E44.304, MTS, Eden Prairie, USA). Traction was applied through the steel wires at a speed of 5 mm/min (Figure 3(a)) until the metal sample was completely pulled out of the patellar tendon (Figure 3(b)). The maximum load during the test is recorded as the failure load.

### 2.7. Statistical Analysis

The statistical analyses were performed using SPSS 22.0 software (IBM Inc.). The statistical significance of the differences among more than three groups was determined by one-way ANOVA, and the significance of the differences among multiple groups under multiple conditions was determined with two-way ANOVA followed by Tukey's test for multiple comparisons. The results are presented as the means ± SD. Differences reached statistical significance at ^∗^*P* < 0.05, ^∗∗^*P* < 0.01, ^∗∗∗^*P* < 0.001, and ^∗∗∗∗^*P* < 0.0001.

## 3. Results

### 3.1. Characterization of the Porous Titanium Implants

Four types of titanium implants were successfully manufactured by SLM and were ready to use. The overall appearance of SLM manufactured titanium implants is shown in Figure S1.

#### 3.1.1. Porosity

We aimed for a porosity of 85% for the manufactured porous implants. The apparent volume of the specimens was 34.54 mm^3^, and 5 specimens were weighed in each group. The porosity of the manufactured implants was 78.12% (SD = 0.45) for the Ti300 implant, 82.76% (SD = 0.54) for the Ti500 implant, and 83.01% (SD = 0.58) for the Ti700 implant (Table 1).

^a^Mean ± standard deviation (SD; %), calculated from the weight and the apparent volume of the specimens. ^b^Mean ± SD (*μ*m), calculated from the microfocus X-ray computed tomography (micro-CT) data. ^c^Surface area and volume of the specimens calculated from micro-CT data.

#### 3.1.2. Microfocus X-Ray Computed Tomography-Based Structural Analysis

Images of the microstructures of the porous implants obtained via micro-CT revealed that all four specimens had remarkable irregularities in the surface with small pores inside the material structure as a result of the incomplete melting of the titanium powder. The pore and material structure shapes were well controlled as designed in all the specimens. The pores were well connected, with no metal powder remaining in specimens Ti500 and Ti700, but a few powders remained in specimen Ti300 due to its narrower aperture (Figures 1(e)–1(h)).

The average pore sizes, average material strut sizes, and specific surface areas of each implant are shown in Table 1. The pore volume distributions are shown in Figure 1(i). Eighty percent of the pores had a pore size between 192 and 264 *μ*m for the Ti300 implant, between 288 and 480 *μ*m for the Ti500 implant, and between 336 and 744 *μ*m for the Ti700 implant. The material strut size was larger than designed due to the incomplete melting of the metal powder; so, the porosity was smaller than designed.

#### 3.1.3. Scanning Electron Microscopy Observations

SEM images of the specimens are shown in Figures 1(j)–1(q). All four groups of specimens were well reproduced as designed. The Ti300, Ti500, and Ti700 specimens had the same braided texture with regularly distributed pores (Figures 1(k)–1(m)). Under high magnification, the surfaces appeared mildly wavy in all four groups with partially melted powder without high peaks and scarped flanks (Figures 1(n)–1(q)). The Ti300 implant had a remarkably higher rate of incomplete melted powders and a much rougher surface than the other implants.

### 3.2. Histological Examination of the Patellar Tendon with the Implants

All the rabbits tolerated the surgical procedure well. No infections of the surgical site or systemic adverse reactions were observed. No body weight loss was observed in the experimental groups compared with the sham group (Figure S2). No dislocation of the implant or adverse reactions such as inflammation or foreign body reactions on or around the implant was observed during specimen collection.

#### 3.2.1. HE Staining and Histological Score

Histological images representative of each sample group are shown in Figures 2(e)–2(m) (at 4, 8, and 12 weeks), and low-magnification images for an overall view are shown in Figure S3. At week 4, very few fibers (“^∗^” in Figures 2(e)–2(m)) were observed in the pores of the Ti300 and Ti500 implants; more fibers were observed in the Ti700 group than in the Ti300 and Ti500 groups but with few nucleated cells. At week 8, fibers filled the pores almost completely in the Ti500 and Ti700 groups, and quite a few fibers could be seen in the pores in the Ti300 group. The Ti500 group had more compact and parallel arranged collagen fibers than the Ti700 group. In contrast, the fibers in the Ti300 group seemed to be tattered and not orderly. In terms of cellularity, the Ti300 implant had fewer cells (arrows in Figures 2(i)–2(m)) in the pores than the other implants due to less tissue growth. Vascularization was observed in the Ti500 and Ti700 groups, and more arteries (“A” in Figures 2(i) and 2(l)) could be seen in the Ti500 group. At week 12, fibers filled the pores in all the groups, and the Ti500 and Ti700 implants exhibited better collagen organization and better cell alignment. More cells were observed in the Ti300 group in week 12 than in week 8, but the organization exhibited by these implants was not as good as that exhibited by the other implants. Vascularization of all three groups in week 12 seemed similar to that observed in week 8. To quantify the histological findings, the HE-stained sections were scored in terms of extracellular matrix, cell morphology, and vascularization using the modified histological score system [38] presented in Table S2. All the scores were significantly lower in the Ti300 group than in the other groups at each time point, and at week 12, the extracellular matrix score and total score of the Ti500 group were 6 ± 0 and 13.67 ± 0.58, respectively, which were significantly higher than those of the Ti700 group. The detailed scores are shown in Figures 2(n)–2(q).

#### 3.2.2. Sirius Red Staining and Collagen Remodeling in the Pores

**Figure 4:**
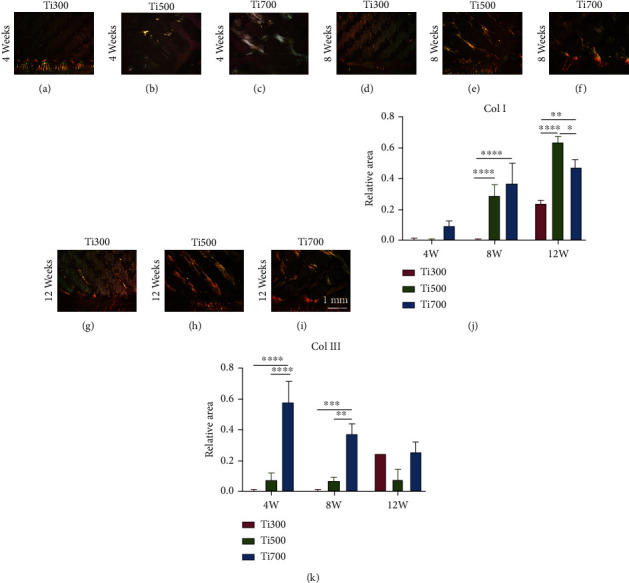
Improvement in collagen composition. (a)–(i) Representative images of Sirius red-stained sections. (j) Statistical analysis of the type I collagen area in pores (*n* = 5). (k) Statistical analysis of the type III collagen area in pores (*n* = 5).

Images of Sirius red-stained sections are shown in Figures 4(a)–4(i). Observed under polarized light microscopy, collagen type I appears to be red or orange, and collagen type III is green. We calculated the area of collagen type I and collagen type III in the pores of each group (Figures 4(j) and 4(k)) and found that collagen type I increased gradually with time. The area percentage of type I collagen in the Ti500 group at week 12 was the highest (63.90% ± 3.41%) and was significantly higher than that in the Ti300 and Ti700 groups (*P* < 0.0001 and =0.0334, respectively). The Ti500 group tended to produce less type III collagen at different time points.

### 3.3. Biomechanical Test of the Patellar Tendon with the Implants

The detachment failure loads for each group at week 12 after implantation are summarized in Figure 3(c). The failure loads of the solid, Ti300, Ti500, and Ti700 groups were 17.03 ± 3.66 N, 44.46 ± 11.26 N, 101.62 ± 13.69 N, and 54.66 ± 11.17 N, respectively. The failure load of the Ti500 group was significantly higher than that of the other groups. Representative force-displacement curves of each sample group in the biomechanical test are shown in Figure S4.

## 4. Discussion

In this study, we manufactured porous Ti6Al4V implants with an intended porosity of 85% and pore sizes of 300, 500, and 700 *μ*m (designated Ti300, Ti500, and Ti700, respectively) by SLM and investigated the optimal pore size for tendon integration by in vivo experiments. We confirmed that the porous structures were reproduced as designed with no gross defects, and the pore sizes were evaluated as 261, 480, and 678 *μ*m. The Ti500 implant exhibited a better histological performance and collagen composition than the other two porous implants at week 12. In addition, the fixation ability of the Ti500 implant was remarkably higher than that of the other implants.

Many studies on porous materials have been conducted recently, mainly on osseointegration [28, 40]. Porous materials provide a larger specific surface area and access for cells, oxygen, and nutrients to improve the biocompatibility of materials and promote tissue integration [22]. With the in-depth study of porous materials, increasing attention has been given to the effect of porous materials on the integration of soft tissues [26–29, 41]. However, previous studies either used porous material with a poorly controlled porous structure [41] or nonweight bearing tissue, such as corium [27, 29] and fascia [26, 28]. Therefore, the most suitable structure for tendon fixation has been unclear.

Titanium and its alloys have long been known to be excellent biocompatible metals with good tolerance and have been used in orthopedic and dental surgery for decades [42], which makes these materials the obvious choice for the exploration of appropriate porous structures for tendon fixation. We referred to research on bone integration to design the possible pore structures used in the current study. In general, the porous structure facilitates cell adhesion, proliferation, and differentiation and provides a rich interface bonding area for blood vessel formation and tissue ingrowth [43, 44]. For an ideal orthopedic porous implant, the porosity should be higher than 40%-50% [28, 45], and higher porosity means a higher specific surface area for tissue fixation. Moreover, the internal interconnection of the pores is necessary for oxygen and nutrient exchange [46, 47]. The best design regarding the pore size is unclear. Chen et al. found that a scaffold with a pore size of 500 *μ*m showed the best bone ingrowth in a rat model [28], while Li et al. claimed that 300-400 *μ*m is the best pore size for goat metatarsus defects [43]; other studies on the treatment of bone defects in rabbits concluded that the best pore sizes were 400 *μ*m and 600 *μ*m, respectively [16, 34]. According to previous studies, the pore size should be between 300 and 600 *μ*m.

Cell differentiation is caused by mechanical biological stimulation, and the morphology of the scaffold plays a key role in controlling its fate and hence regenerating tissue [48]. The curvature of the surface on which cells reside in particular has been demonstrated to play a fatal role in the tissue regeneration rate [49, 50]. Therefore, a physiological porous structure may promote tendon fixation. Rony et al. compared implants with trabecular microarchitecture and purely geometric microarchitecture and reported that the former did not show better osseointegration [40]. Therefore, it seems irresponsible to simply imitate the trabecular bone or tendon structure. Previous research on geometric structure provided some insight, namely, that the cells and tissue tend to reduce the curvature as much as possible [32, 50–52]. Coincidentally, the mean curvature of trabecular bone is close to zero [33]. We tried to find a porous structure with minimal surfaces and chose the geometric model of TPMSs, which has a mean curvature of zero that can be indefinitely extended in three periodic directions [49]. According to all the research above, we used the porous structure of TPMSs with three pore sizes (300, 500, and 700 *μ*m) and with a constant porosity of 85% in our study.

In addition to the structural design, the production of scaffolds is an important aspect in the fabrication process and includes accurate control of the pore size, pore distribution, and pore interconnectivity. AM is suited to the manufacture of porous titanium implants with a precisely controlled pore size, pore distribution, and pore interconnectivity. SLM technology and electron beam melting (EBM) technology are typical processes for metal additive manufacturing [53, 54]. SLM can produce specimens with higher machining accuracy and smoother surfaces [55], hence reducing the design of porous structures more precisely. Moreover, the mechanical strength and fatigue strength of the samples produced by SLM are better than those produced by EBM with fewer internal defects [56, 57].

Characterization of the porous specimens proved the reliability of the SLM process. Micro-CT analysis of the specimens showed minor deviation in the morphological parameters. Although the postprocessing of micro-CT data is a subjective process [58], appropriate postprocessing can reveal the morphology of a specimen. However, there were some variations between our design and the measured pore parameters. The pore size and porosities were smaller than those designed in all three groups, and the parameters in the Ti300 group had the largest difference from the design. These findings may be due to the incompletely melted metal powder on the periphery of the material strut. Ti300 group have the minimum porosity in all three groups could result from its high surface area on which more incompletely melted powder was adhered. In addition, the material strut appeared rough with the incompletely melted metal powder. The mild undulant surfaces were considered a suitable matrix that can trigger the attachment and proliferation of cells [16] and provide better biocompatibility than polished titanium.

To reduce the variables in our test, we chose animal models from a previous study in which whole implants were embedded in the rabbit patellar tendon. In comparison with experimental models in previous studies of tendon attachment to metal, our model was more feasible in that implant preparation and operative methods were simple and easy and could be more repeatable. First, compared to fixation with a washer or plate [18, 59], our design could normalize the interface area between the tendon and the implant as the porous area of the implant and prevent false positives due to larger contact areas. The solid part on top of the specimen (Figures 1(a)–1(d) and Figure S1) was designed to normalize the fixation area in the mechanical tests. Second, compared with fixation with screws, the in-tendon model reduced the influence of bone marrow-derived stem cells [59, 60]. Third, a simpler surgical procedure reduced the operation time and bleeding, and the interface was cleaned due to its low blood supply, similar to tendons. Some limitations of the animal model should be acknowledged, which are that the initial fixation and the implantation method of the current model did not imitate clinical conditions in a nonload-bearing condition. Further study under load-bearing conditions will be necessary.

Regarding the biological effects of the porous structures, the Ti500 group had advantages in collagen arrangement and collagen composition. The spaces in the scaffolds played an important role in tissue ingrowth and vascularization. Unlike in vitro cell proliferation, which demands a large specific surface area, in vivo tissue ingrowth requires an appropriate porous characteristic for cell migration and exchange of nutrients. Notably, 300 *μ*m is apparently too small to support enough ingrowth in our study. As previously demonstrated, elongated cell morphology and oriented cell arrangement are conducive to the ordered deposition of the extracellular matrix [61], which might be the reason why the Ti500 group had a better fiber alignment than the Ti700 group. Moreover, a better collagen composition in the Ti500 group could also result in the appropriate mechanical biological stimulation from the right pore characteristics.

Remarkably, the Ti500 and Ti700 groups exhibited favorable vascularization. As shown in previous studies, the space in the scaffolds has an important role in vascularization [28, 43]. The vascularization in pores further promotes the growth of tissue. Previous studies have suggested that porous scaffolds with a pore size > 300 *μ*m induced angiogenesis [62], and that there was no marked increase in the extent of vascularization with a further increase in pore size above 400 mm [63], which is consistent with our findings.

The aim of finding the optimal pore structure is to accelerate tissue integration and enhance the fixation strength. Not surprisingly, the biomechanic results revealed that the Ti500 group had the highest failure load of approximately 102 N, which was much higher than that of the Ti300 and Ti700 groups. Although a higher specific surface area contributes to tissue integration, the larger amount of tissue in the pore and more regular collagen arrangement clearly contributed more to the improved biomechanics. Because of the differences in the contact area, fixing method, and duration of the test, it is difficult to compare the biomechanical results between different studies. Compared with 12.9 N used in a similar animal model at 8 weeks postoperatively [19], a well-designed porous structure showed a higher fixation capacity than sintered porous titanium implants.

The experimental model used in this study has some limitations. First, to minimize the interference of different unit cell types on tissue integration, only one of them was introduced in the design of porous scaffolds. To investigate the application potential in orthopedics, architectures containing more unit cell types should be tested in the future. Second, as mentioned above, the initial fixation did not imitate clinical conditions and was applied to a nonload-bearing animal model. Studies with a load-bearing animal model need to be explored in the future. However, our study revealed the effect of pore size on the fixation of soft tissue on porous structures, achieved notable histological and biomechanical results, and provided insights for future research.

## 5. Conclusions

Our study confirmed that a Ti6Al4V implant with a diamond-like porous structure with a triply periodic minimal surface pore size of 500 *μ*m promoted tendon integration, providing a promising design for tendon insertion into prostheses.

## Figures and Tables

**Table 1:** Porosity and microfocus X-ray computed tomography-based three-dimensional structural analysis.

Group	Porosity (%)^a^	Pore size^b^	Material strut size^b^	Specific surface area (/mm)^c^
Ti300	78.12 ± 0.45	261.16 ± 5.41	360.1 ± 1.13	6.19 ± 0.03
Ti500	82.76 ± 0.54	480.15 ± 3.41	446.6 ± 12.87	3.77 ± 0.07
Ti700	83.01 ± 0.58	677.54 ± 7.95	456.63 ± 9.34	3.42 ± 0.05

## Data Availability

The datasets generated and analyzed during the present study are available from the corresponding author upon reasonable request.

## Supplementary Materials

Supplementary Materials The following figures and tables are provided in supplemental file: Figure S1: overall appearance of the SLM manufactured porous titanium implants. Figure S2: statistical analysis of rabbit weight after surgery. Figure S3: overall view of the HE-stained sections of each sample group. Figure S4: representative force-displacement curve of each sample group. Table S1: physical and chemical properties of Ti6Al4V powder. Table S2: histological scoring system.
