# Supplementary material for: Effect of Pore Size of Porous-Structured Titanium Implants on Tendon Ingrowth
Source: Appl Bionics Biomech. 2022 Apr 25;2022:2801229. doi: 10.1155/2022/2801229 (PMC9061050; doi:10.1155/2022/2801229)
Supplement: Supplementary Materials — The following figures and tables are provided in supplemental file: Figure S1: overall appearance of the SLM manufactured porous titanium implants. Figure S2: statistical analysis of rabbit weight after surgery. Figure S3: overall view of the HE-stained sections of each sample group. Figure S4: representative force-displacement curve of each sample group. Table S1: physical and chemical properties of Ti6Al4V powder. Table S2: histological scoring system. [file 2801229.f1.docx]

Additional File for

**Effect of pore size on tendon ingrowth into porous titanium implants**

Yupeng Guo, Fei Liu, Xuting Bian, Kang Lu, Pan Huang, Xiao Ye, Chuyue Tang, Xinxin Li, Huan Wang, and Kanglai Tang

**This additional file includes:**

Fig. S1 Overall appearance of the SLM manufactured porous titanium implants.

Fig. S2 Statistical analysis of rabbit weight after surgery.

Fig. S3 Overall view of the HE-stained sections of each sample group.

Fig. S4 Representative force-displacement curve of each sample group.

Table S1 Physical and chemical properties of Ti6Al4V powder.

Table S2 Histological scoring system.

**Supplementary Figure 1 Overall appearance of the SLM manufactured porous titanium implants.**


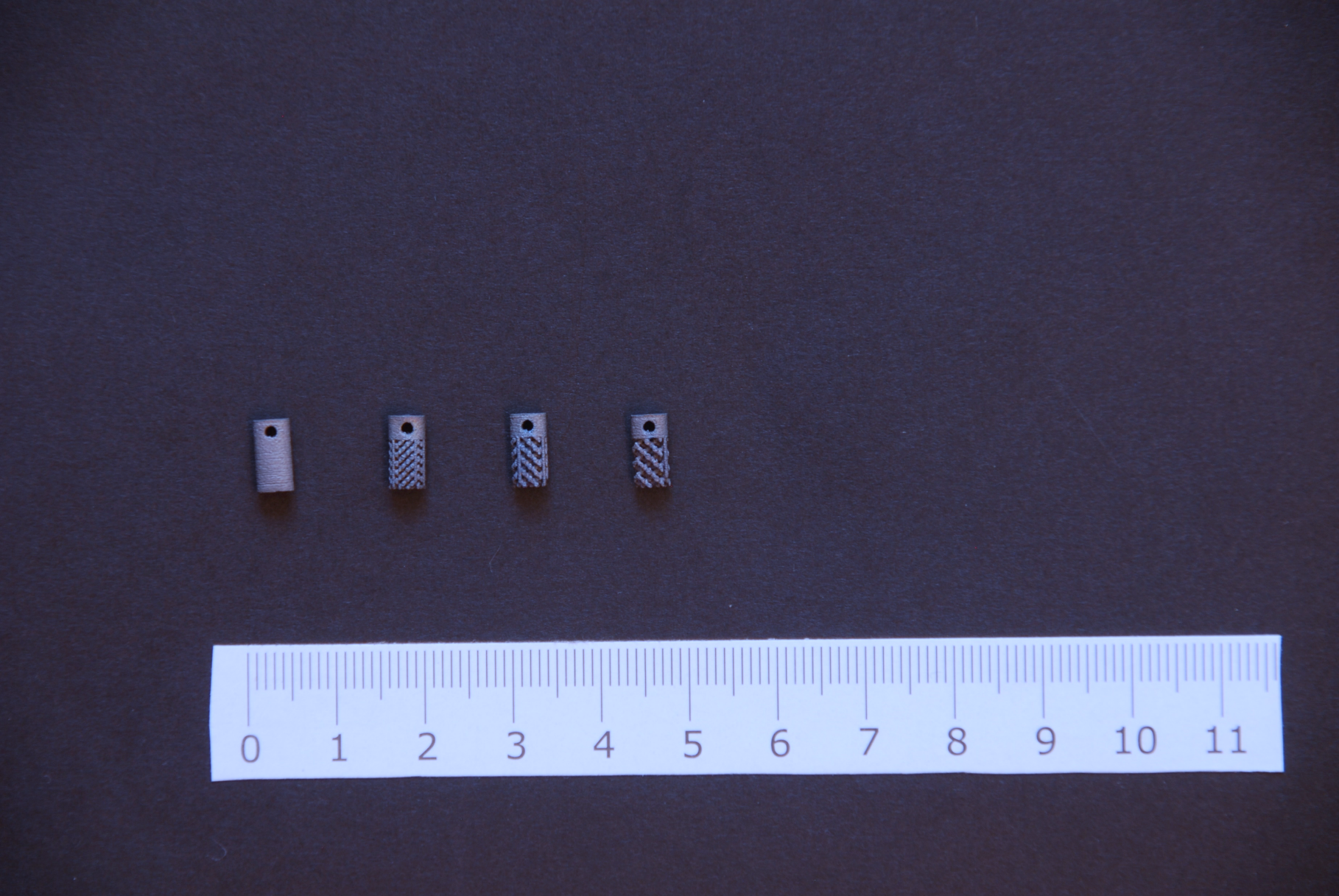


**Fig. S1** Appearance of the SLM manufactured porous titanium implants: From left to right are the solid, Ti300, Ti500 and Ti700 implants. Scale unit: cm.

**Supplementary Figure 2 Statistical analysis of rabbit weight after surgery.**


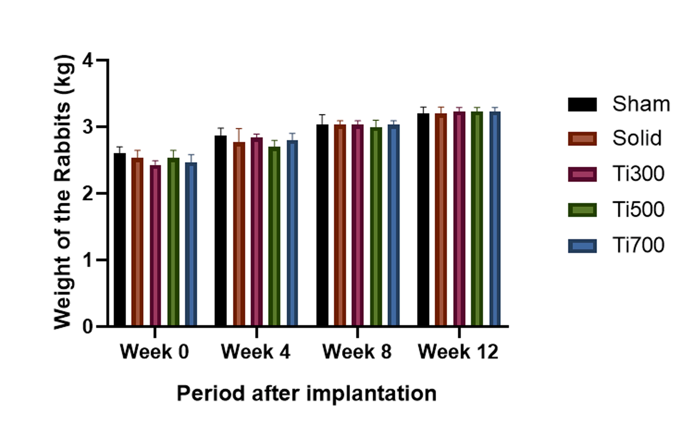


**Fig. S2** Statistical analysis of rabbit weight after surgery: **black column,** sham group; **brown column,** solid group**; purple column,** Ti300 group**; green column,** Ti500 group; **blue column,** Ti700 group.

**Supplementary Figure 3 Overall view of the HE-stained sections of each sample group.**


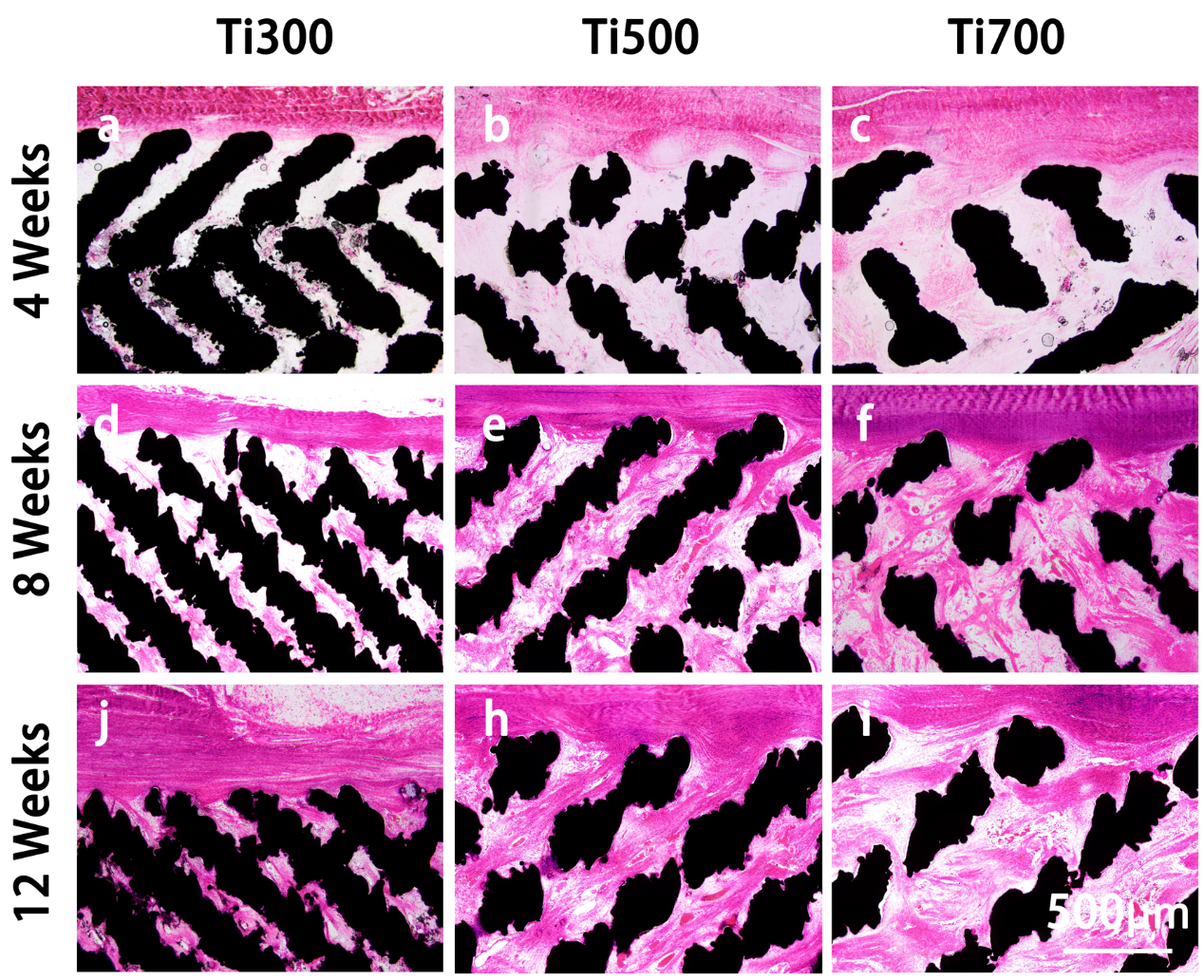


**Fig. S3** HE-stained sections of each sample group at 40×.

**Supplementary Figure 4** **Representative force-displacement curve of each sample group.**


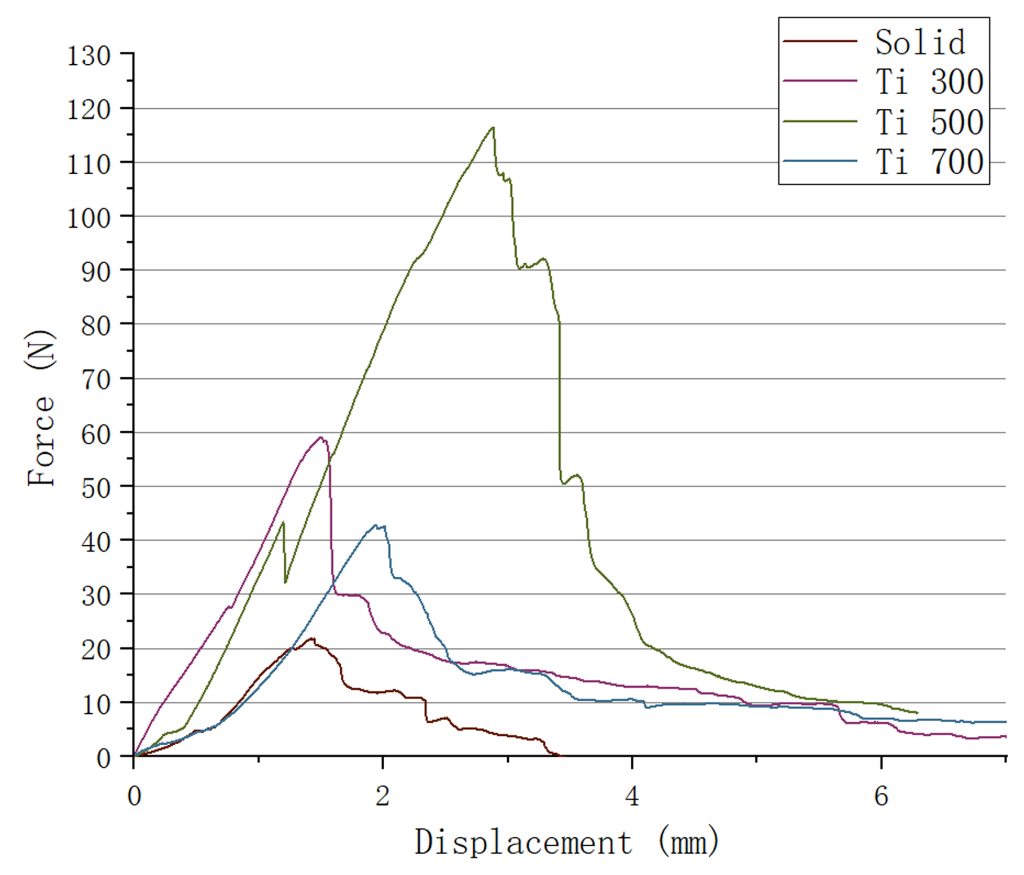


**Fig. S4** Representative force-displacement curve: **Brown line,** solid group; **Purple line,** Ti300 group; **Green line,** Ti500 group; **Blue line,** Ti700 group.

**Supplementary Table 1** **Physical and chemical properties of Ti6Al4V powder**

| **Chemical composition** | | | | |
| --- | --- | --- | --- | --- |
| **Element** | **Content** | | **Element** | **Content** |
| Ti | Other90.06 | | N | 0.025% |
| Al | 5.83% | | Fe | 0.013% |
| V | 3.98% | | C | 0.008% |
| O | 0.083% | | H | 0.0038% |
| **Physical property** | | | | |
| **Particle size (μm)** | | 15-53 | | |
| **Mobility (s/50 g)** | | 36 | | |
| **Loose density (g/cm3)** | | 2.41 | | |
| **Tamping density (g/cm3)** | | 2.85 | | |

**Supplementary Table 2 Histological scoring system**

| Group | Evaluated parameters | Points |
| --- | --- | --- |
| *Extracellular matrix* | *Extracellular matrix (ECM) morphology* |  |
|  | Long continuous fiber | 3 |
|  | Ruptured fiber less than 1/3 | 2 |
|  | Ruptured fiber less than 2/3 | 1 |
|  | Broken and completely discontinuous fibers | 0 |
|  | *Extracellular matrix (ECM) organization* |  |
|  | Compact and parallel arranged | 3 |
|  | Somewhat loose, wavy | 2 |
|  | Looser with intersecting fibers | 1 |
|  | Loosely composed, not orderly | 0 |
| *Cells* | *Cellularity* |  |
|  | Increased cell density | 2 |
|  | Physiological or mild decreased cell density | 1 |
|  | Few cells or no visible cells | 0 |
|  | *Cell alignment* |  |
|  | Uniaxial | 2 |
|  | Areas of irregularly arranged cells (10-50%) | 1 |
|  | More than 50% of cells with no uniaxial alignment | 0 |
|  | *Cell nucleus morphology* |  |
|  | Predominantly elongated, heterochromatic cell nuclei (tenocytes) | 2 |
|  | 10-30% of the cells possess large, oval, euchromatic or polymorph heterochromatic nuclei | 1 |
|  | Predominantly larger, oval, euchromatic or polymorph, heterochromatic nuclei | 0 |
| *Vascularization* | *Vascularization in pores* |  |
|  | Hypervascularized (increased numbers of small capillaries with arterioles) | 2 |
|  | Hypovascularized (increased numbers of small capillaries) | 1 |
|  | Nonvascularized | 0 |
